# Performance Evaluation of MICP in Crack Repair: Strength and Durability Enhancement Using Different Bacterial Strains

**DOI:** 10.3390/ma19153356

**Published:** 2026-08-06

**Authors:** Michelle Tinotenda Nyambi, Chunhua Lu, Wenshuo Li, Weiqi Zhang

**Affiliations:** School of Intelligent Science and Engineering, Jiangsu University, Zhenjiang 212013, China

**Keywords:** microbially induced calcium carbonate precipitation (MICP), crack repair, tensile strength, durability performance, microstructure analysis

## Abstract

Concrete is fundamentally susceptible to cracking, which creates pathways for aggressive agents, accelerating reinforcement corrosion and reducing service life. Traditional repair methods are ineffective for micro-cracks and generate volatile compounds. Microbially induced calcium carbonate precipitation (MICP) offers a sustainable bio-based alternative that catalyses in situ calcium carbonate (CaCO_3_) precipitation within cracks, sealing pathways. This study compares two MICP repair systems consisting of *Sporosarcina pasteurii* (SP, ureolytic) and *Bacillus mucilaginosus* (BM, non-ureolytic), utilising a dual-viscosity-modifying-agent (VMA) system comprising Welan Gum and Attagel 50 (WA). The repair treatments were applied externally on cracked concrete specimens across crack widths of 0.10–0.80 mm for 16 days. Crack repair effectiveness was evaluated through splitting tensile strength, capillary water absorption, rapid chloride migration (RCM), and X-ray diffraction (XRD). SP + WA exhibited high performance trends compared with BM + WA, achieving tensile strength retention of 59.57–68.95% vs. 56.68–67.15%, capillary water absorption recovery ranges of 58.36–64.81% vs. 51.71–58.02%, chloride resistance recoveries of 63.7–82.1% vs. 50.5–60.0%, and *D*_RCM_ recoveries of 57.35–77.68% vs. 46.04–61.00% relative to intact controls. Durability recovery efficiency decreased with increasing crack width. XRD confirmed calcite as the sole CaCO_3_ polymorph in both systems, with sharper peaks in SP + WA indicating differences in calcite crystal characteristics, while BM + WA produced broader peaks similar to a nanocrystalline CaCO_3_ structure. These findings demonstrate that the dual-VMA-assisted MICP approach potentially improves mechanical and durability properties in cracked concrete, with ureolytic SP demonstrating better repair performance trends.

## 1. Introduction

Concrete is the most widely produced construction material worldwide, valued for its compressive strength and versatility in infrastructure. Despite these advantages, concrete is fundamentally susceptible to cracking, which may initiate during the plastic state through plastic shrinkage or in the hardened state due to mechanical overloading, thermal expansion, chemical reactions, and cyclic environmental exposure [1,2]. Cracks create pathways for moisture, chloride ions, and carbon dioxide ingress, accelerating reinforcement corrosion and reducing both load-carrying capacity and service life [3,4]. The consequences extend beyond structural risk; frequent inspection, repair, and premature replacement impose substantial economic and environmental burdens [5]. Traditional repair methods, including organic coatings, cementitious grouting, and epoxy injection, are often ineffective for micro-cracks and hard-to-reach locations, while synthetic materials generate volatile gases that compound their environmental footprint [6,7]. When deterioration progresses unchecked, structural elements require demolition and reconstruction, generating enormous volumes of waste while consuming fresh raw materials [8,9].

The cement and concrete industry contributes approximately 8% of global CO_2_ emissions, releasing roughly 1 kg of CO_2_ per kilogram of cement produced, with 3–4 billion tonnes emitted annually [10,11], reflecting its indispensable role in modern civilisation and underscoring the construction sector’s substantial role in climate concerns. The United Nations Sustainable Development Goals explicitly call for sustainable cities and responsible material consumption [12,13], while China’s 2020 commitment to peak emissions by 2030 and achieving carbon neutrality before 2060 further underscores the urgency [14,15,16]. These imperatives persuade the development of bio-based, low-carbon concrete repair technologies that address structural deterioration while reducing environmental impact. Biochar, a carbon-negative material produced through biomass pyrolysis, has demonstrated significant potential for atmospheric carbon sequestration while enhancing the mechanical properties of engineered substrates [17,18,19], and zinc-modified biochar catalysts have shown enhanced thermal behaviour for sustainable energy applications [20], illustrating the broader paradigm of bio-based materials. Proton exchange membrane fuel cells and hybrid energy management systems have further demonstrated improved electrical efficiency and reduced hydrogen consumption in industrial applications [21,22], reinforcing the transition toward low-carbon technologies across sectors.

The convergence of these developments creates a compelling rationale for microbially induced calcium carbonate precipitation (MICP) as a biologically derived repair technology that operates within the same sustainability framework. MICP exploits microbial metabolic activity to catalyse the in situ precipitation of CaCO_3_ within cracks, physically sealing pathways and restoring mechanical continuity without synthetic compounds or hazardous by-products [23]. As a biomineralisation process, microorganisms convert soluble calcium compounds into insoluble CaCO_3_ through enzymatic metabolic reactions, with precipitation mechanisms, bacterial material interactions, and characterisation protocols having been extensively reviewed in the literature [24]. The predominant concrete repair pathway is urea hydrolysis, where ureolytic bacteria catalyse urea breakdown into carbonate and ammonium ions for CaCO_3_ precipitation at cell surface nucleation sites [25,26,27], a process enhanced by Ca^2+^ supplementation that protects cells under alkaline stress [28]. Environmental remediation applications have further demonstrated the versatility of hybrid bio–nano approaches, with nanoscale zero-valent iron composites achieving effective heavy metal removal [29]. The carbonic anhydrase pathway employed by non-ureolytic bacteria such as *Bacillus mucilaginosus* catalyses CO_2_ hydration to produce bicarbonate ions that react with calcium, avoiding the ammonium by-product of ureolysis, though generally at lower precipitation rates [23,30,31]. The distinction between these two pathways has significant scientific and practical implications that remain inadequately resolved. Early studies demonstrated that sprayed bacterial suspensions produced dense calcite layers verified by SEM and EDS, reducing capillary water absorption and air permeability [32], with subsequent work reporting compressive strength increases of 36.7% and water absorption reductions of 20% [33]. Optimised MICP in mortar promoted nano-calcite formation that filled gel pores and improved both capillary absorption resistance and compressive strength [34], while Tan et al. [35] showed that MICP altered mineral phases near crack interfaces, with C-S-H serving as a secondary calcium source. Another study focused on aerated concrete’s phosphorus adsorption–desorption behaviour [36], demonstrating its dual structural–environmental functionality, a principle extendable to MICP self-healing.

Application methods include injection, immersion, diffusion, spray, and combined strategies. Tittelboom et al. [37] demonstrated that the injection of 0.1 mm cracks achieved repair quality comparable to epoxy resin. Lu et al. [38] compared injection and diffusion application methods and found that diffusion via sponge strips achieved 79.7% permeability and 60.9% chloride resistance improvements, outperforming injection via extended residence time for greater CaCO_3_ accumulation. The spray method produced a 55.7% decrease in water absorption, a 45.1% reduction in permeability, and an 8.7% reduction in electrical conductivity [39], with repeated application enhancing compressive strength by 31.69% [40]. Carrier systems protect bacterial spores from mechanical stresses and alkaline environments [41]; sodium alginate-encapsulated spores achieved 100% survival and reduced 0.28 mm cracks to 0.02 mm [42], while microcapsule-embedded spores achieved healing rates of 48–80% [43]. Reviews on MICP for stabilisation have examined mineralisation mechanisms and engineering applications. Viscosity-modifying agents (VMAs) have been introduced in MICP crack repair to improve bacterial retention by increasing suspension viscosity under resting conditions while maintaining flowability during application. Welan gum, a microbial polysaccharide, is widely used as a rheology modifier due to its shear-thinning behaviour and water retention capacity [44]. Studies reported that Welan gum reduced chloride permeability while maintaining approximately 90% of the compressive and flexural strength of control mixtures, with an optimum dosage of approximately 0.05 wt%, beyond which microporosity and strength loss may occur [45,46]. Attagel 50, a purified attapulgite clay, provides complementary thixotropic gelation that improves suspension stability and resistance to gravitational washout, particularly in crack repair applications [47,48]. More recently, a dual-VMA system comprising 0.3% Welan gum and 6% Attagel 50 was shown to enhance bacterial growth, CaCO_3_ precipitation, and suspension retention through synergistic rheological effects [49].

However, this system has only been evaluated with ureolytic bacteria, and comparative assessments of ureolytic and non-ureolytic bacteria repair formulations remain underexplored. To address this gap, the present study compares two MICP repair systems consisting of *Sporosarcina pasteurii* (SP, ureolytic) and *Bacillus mucilaginosus* (BM, non-ureolytic), each formulated within a dual-VMA system comprising Welan Gum and Attagel 50 (WA) for the repair of cracked concrete. Repair effectiveness is evaluated through mechanical recovery, durability performance, and X-ray diffraction characterisation of the resulting CaCO_3_ precipitates.

## 2. Materials and Methods

### 2.1. Concrete Specimens and Crack Induction

All concrete specimens were cast using the mix proportions presented in Table 1, with a water–cement ratio (w/c) of 0.49. Ordinary Portland Cement P.O 42.5 was used throughout. River sand with a fine modulus of 2.7 served as fine aggregate, crushed stone ranging from 5 to 25 mm as coarse aggregate, and tap water for both mixing and curing. Concrete strength grade was designed as C30, and after 28 days of curing, cubic specimens (150 mm × 150 mm × 150 mm) achieved a mean compressive strength of 32.57 MPa.

Three types of concrete specimens were used for crack repair testing, with a total of 18 cylindrical concrete specimens, 150 mm × 300 mm, used for split tensile strength testing, while 24 concrete cubes with dimensions 100 mm × 100 × 100 mm were used for capillary water absorption, and 24 concrete cylinders of size 100 mm × 50 mm for rapid chloride migration tests.

### 2.2. Crack Induction

**Figure 1:**
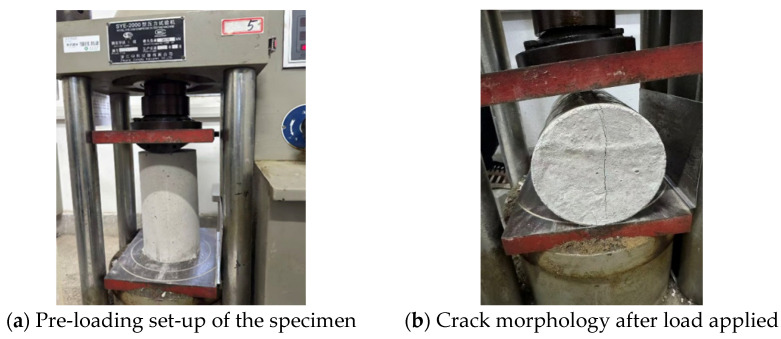
Crack initiation by mechanical loading.

**Figure 2:**
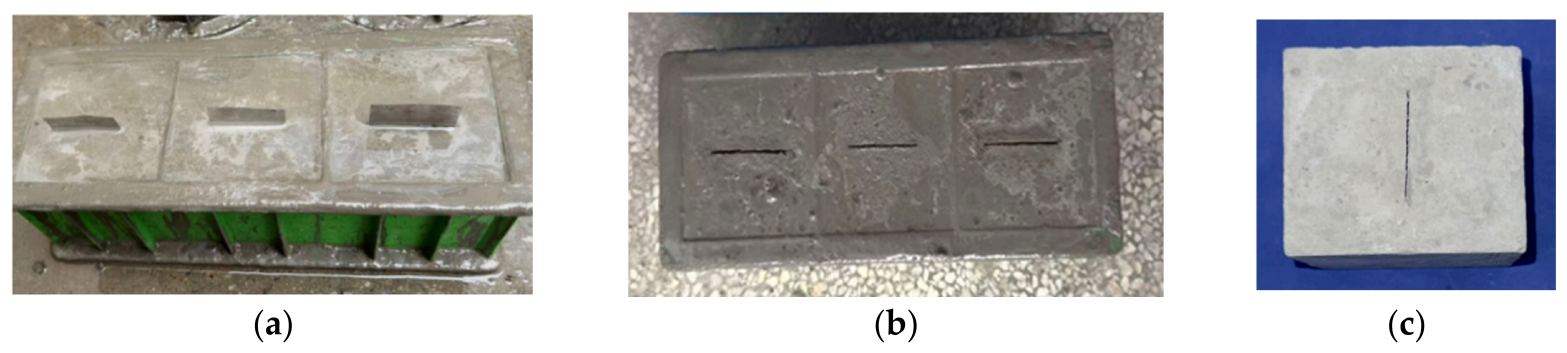
Crack morphology made by steel plates in 100 mm × 100 mm × 100 mm cubic specimens. (**a**) Insertion of steel plates during pouring and casting. (**b**) Steel plates removed 6–8 h post-casting. (**c**) Crack morphology post-curing.

**Figure 3:**
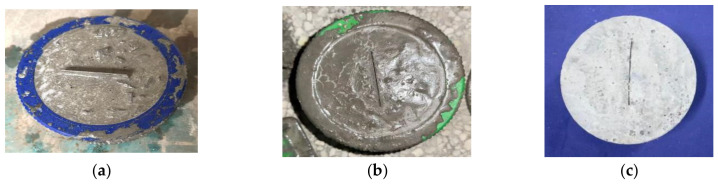
Crack morphology made by steel plates in 100 × 150 mm cylindrical specimens. (**a**) Insertion of steel plates during pouring and casting. (**b**) Steel plates removed 6–8 h post-casting. (**c**) Crack morphology post-curing.

Two methods were used to induce cracks in this study in the following way:

1. By the splitting method, cracks were induced by placing the cylindrical specimen vertically between the loading platens, and a metal platen was placed along the diametric plane to localise the cracking along a defined path; see Figure 1a. The specimen was subjected to a controlled diametric compressive load of approximately 0.03 MPa using a SYE-2000 compression testing machine (Zhejiang ZhongKe Instrument Co., Ltd., Shaoxing, China) until a visible crack propagated; loading was then immediately halted. Two crack width groups of 0.05–0.10 mm and 0.10–0.20 mm were established using this method. Figure 1b shows the resulting diametrical crack formed after loading.

2. The steel plate insertion method was used as another method for crack initiation in cubic and cylindrical specimens of dimensions 100 mm × 100 mm × 100 mm and 100 mm × 50 mm. Steel plates (50 mm height; thicknesses of 0.1, 0.3 and 0.5 mm) were inserted at the centre of the specimen during casting. Plates were removed 6–8 h post-casting during the early hardening stage. Measured crack widths ranged from 0.40 to 0.80 mm due to surface irregularities and elastic recovery after the steel plate removal, producing four crack width groups (0.40–0.50, 0.50–0.60, 0.60–0.70, and 0.70–0.80 mm). Figure 2 and Figure 3 present the crack induction method by inserting steel plates into concrete specimens.

As shown in Figure 1, Figure 2 and Figure 3, the two methods produce visually distinct crack morphologies, reflecting their different formation mechanisms. Mechanical loading generates irregular paths with variable roughness and tortuosity, whereas plate insertion yields more uniform apertures with differing wall contact conditions and depth continuity. Due to their distinct geometrical characteristics, repair performance was evaluated within each corresponding test parameter. Crack depth was controlled by steel plate height for durability specimens; however, it was not independently measured for load-induced specimens due to the unpredictable nature of fracture propagation. Hence, crack width was selected as the main metric for assessing repair performance.

For crack repairs, four test series were established: ICS (intact concrete specimen), crack repaired by SP + WA (*Sporosarcina pasteurii* (CGMCC 1.3687, sourced through China Common Microbiome Species Preservation Management Centre, Beijing, China) with Welan Gum and Attagel 50), crack repaired by BM + WA (*Bacillus mucilaginosus* (CICC 22524, sourced through China Industrial Microbial Species Storage Management Centre, Beijing, China) with Welan Gum and Attagel 50), and UCS (unrepaired cracked specimen). ICS served as the control reference baseline representing uncracked concrete in its pristine state. The UCS series served as the lowest performance threshold, representing the condition of cracked concrete prior to any repair, and the UCS was tested against specimens with the largest crack widths in this study.

### 2.3. Growth Media and Culture Preparation

**Figure 4:**
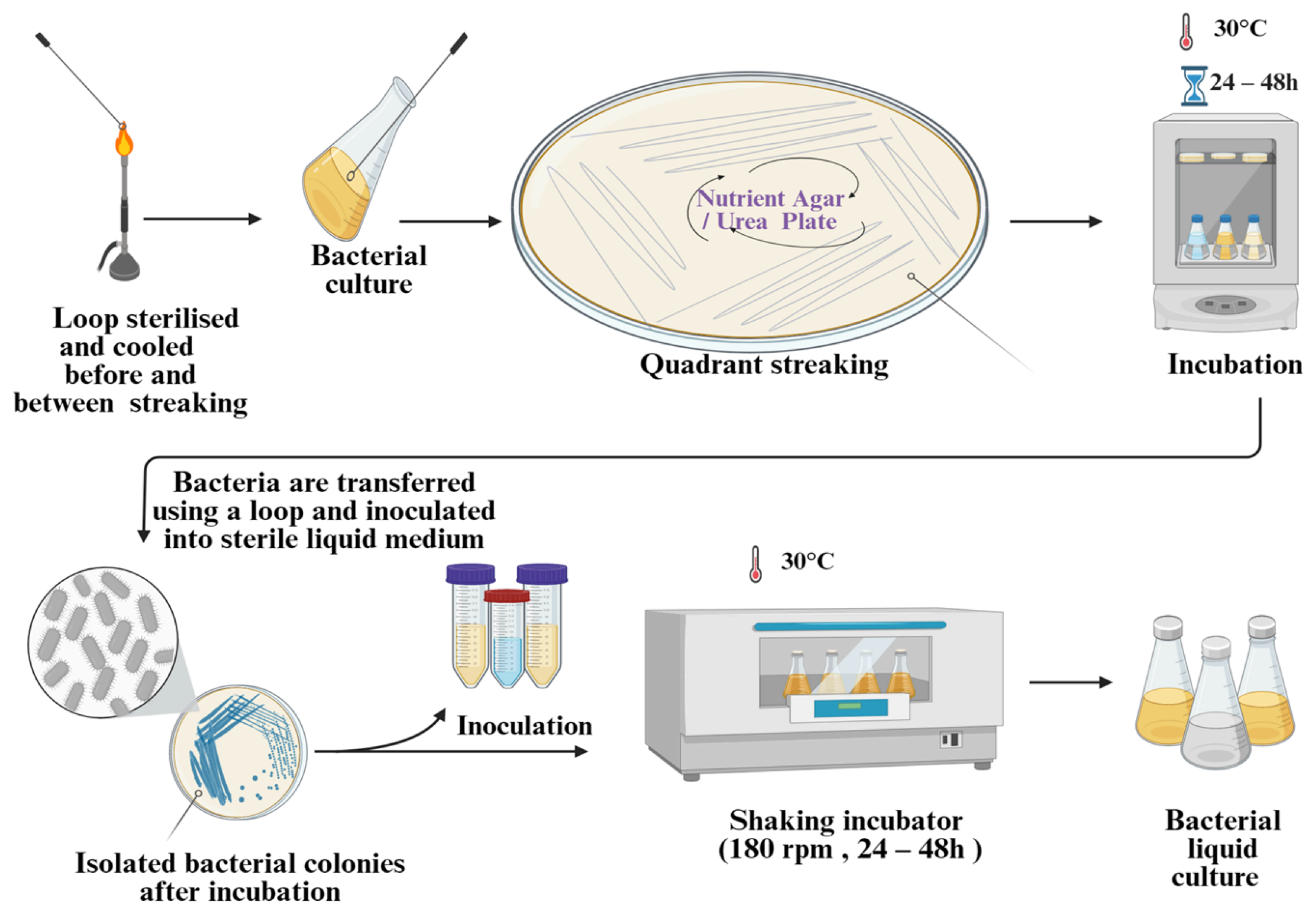
Schematic illustration of bacterial culture preparation.

Bacterial colonies were cultured by plate streaking on solid agar and urea under aseptic conditions, incubated until colony formation for 1–2 days, then transferred to liquid media in a shaking incubator at 30 °C and 180 rpm, with SP cultivated for 36 h and BM for 48 h and stored in the refrigerator. Figure 4 illustrates a summary of the colony growth process to make a fresh bacterial liquid culture. The schematic diagram was created in BioRender (BioRender; https://BioRender.com/mim2utm, accessed on 2 August 2026). The SP growth medium comprised 20 g/L yeast extract, ammonium chloride (NH_4_Cl) 15 g/L, and 0.1 mL/L nickel chloride (NiCl_2_), with a pH range of 9.2–9.3 adjusted using sodium hydroxide (NaOH). The BM growth medium composition consisted of 10 g sucrose, 0.1 g CaSO_4_·2H_2_O, 0.2 g NaCl, 0.2 g MgSO_4_·7H_2_O, 0.2 g K_2_HPO_4_, and 5 g CaCO_3_ in 1 L distilled water, with no pH adjustments.

Welan gum (0.3% *w*/*w*) and Attagel 50 (6%) were dispersed into the two bacterial solutions to form a dual-viscosity-modifying-agent carrier system, following the optimum concentrations established by Feurgard et al. [49]. Each modifier was first prepared separately at double concentration in the bacterial solution and stirred using a magnetic stirrer for 24 h. The two VMAs were blended and stirred for a further 24 h to ensure uniform distribution.

Both bacterial growth media were autoclaved at 121 °C for 30 min before inoculation, and the bacterial solution was then cultivated at 30 °C and 180 rpm. After cultivation, both cultures were assessed, achieving a cell density of 5.67 × 10^8^ cells/mL (OD_600_). Urease activity of SP was measured at 14–18 U/mL using a conductivity metre, and for BM, activity growth was visually monitored through turbidity changes following transfer from a streaked plate and incubation. Its mechanism was based on established reported findings on the carbonic anhydrase-associated pathway [23,30,31]. Fresh cultures were prepared every three days.

Separate cementitious solutions were prepared for each bacterial suspension using 500 mL of deionised water. The SP solution consisted of 1 mol/L calcium acetate and 1 mol/L urea (79.083 g calcium acetate and 30 g urea were used). The BM solution contained 25 mM sodium bicarbonate and 60 mM calcium acetate (1.050 g sodium hydrogen carbonate and 4.745 g calcium acetate).

### 2.4. Crack Repair Based on MICP Treatment Protocol

The mixed MICP + WA suspension was applied externally daily for 16 days. To ensure adequate penetration, the mixture was drawn into a syringe and injected directly into cracks with widths ranging from 0.40 to 0.60 mm at low pressure. The partial immersion method was used for crack widths of 0.60–0.70 mm and specimens subjected to split tensile testing, where a silicone sealant was placed along the crack margins to minimise lateral runoff before pouring the suspension over the fracture surface and entering by gravity flow. Between treatment cycles, specimens were stored at room temperature, uncovered. Table 2 illustrates a summary of concrete specimens showing corresponding test parameters, crack width groups, and total number of specimens used.

### 2.5. Testing Methods

#### 2.5.1. Splitting Tensile Strength

**Figure 5:**
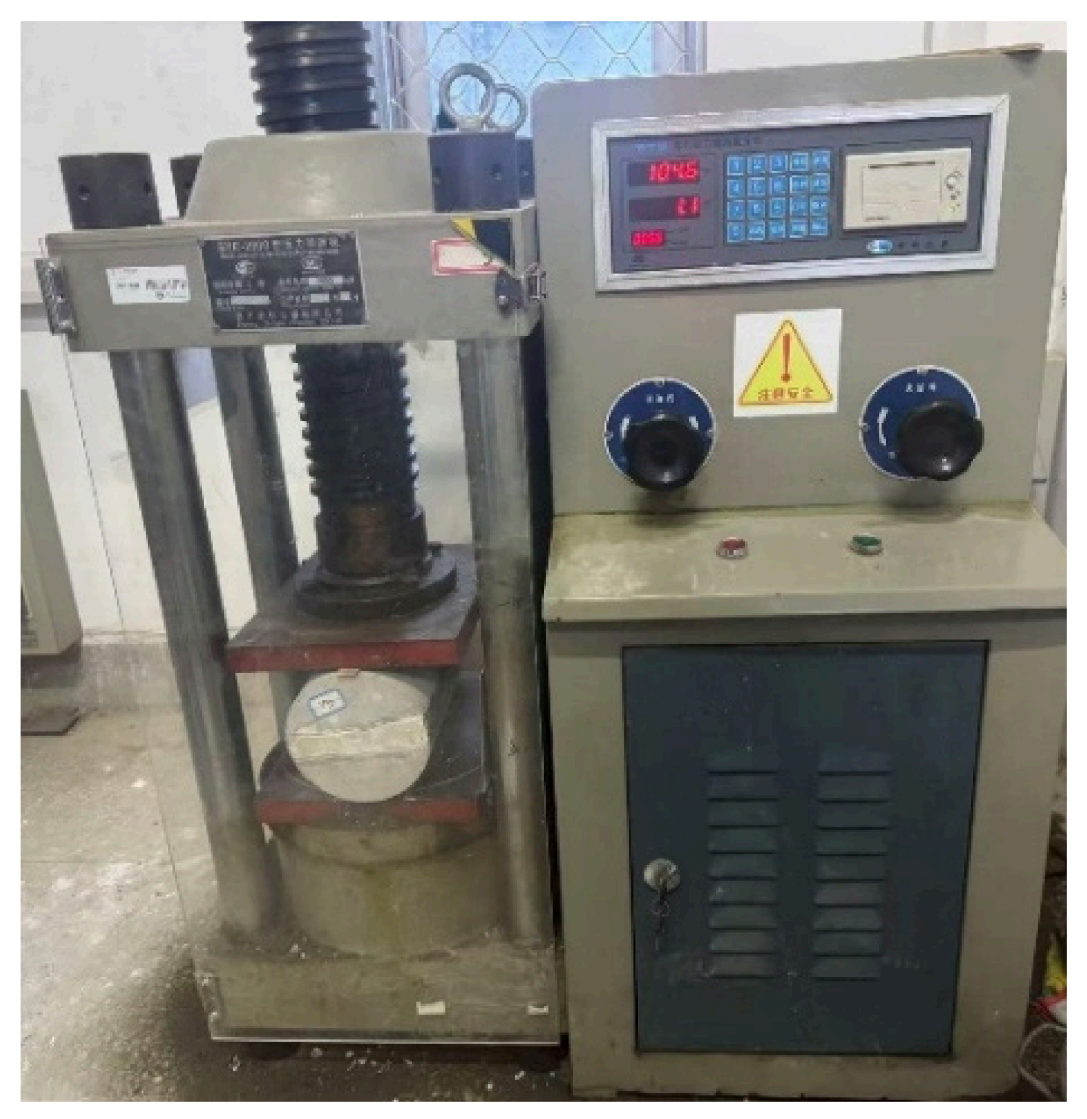
Split tensile testing method after crack repair.

**Figure 6:**
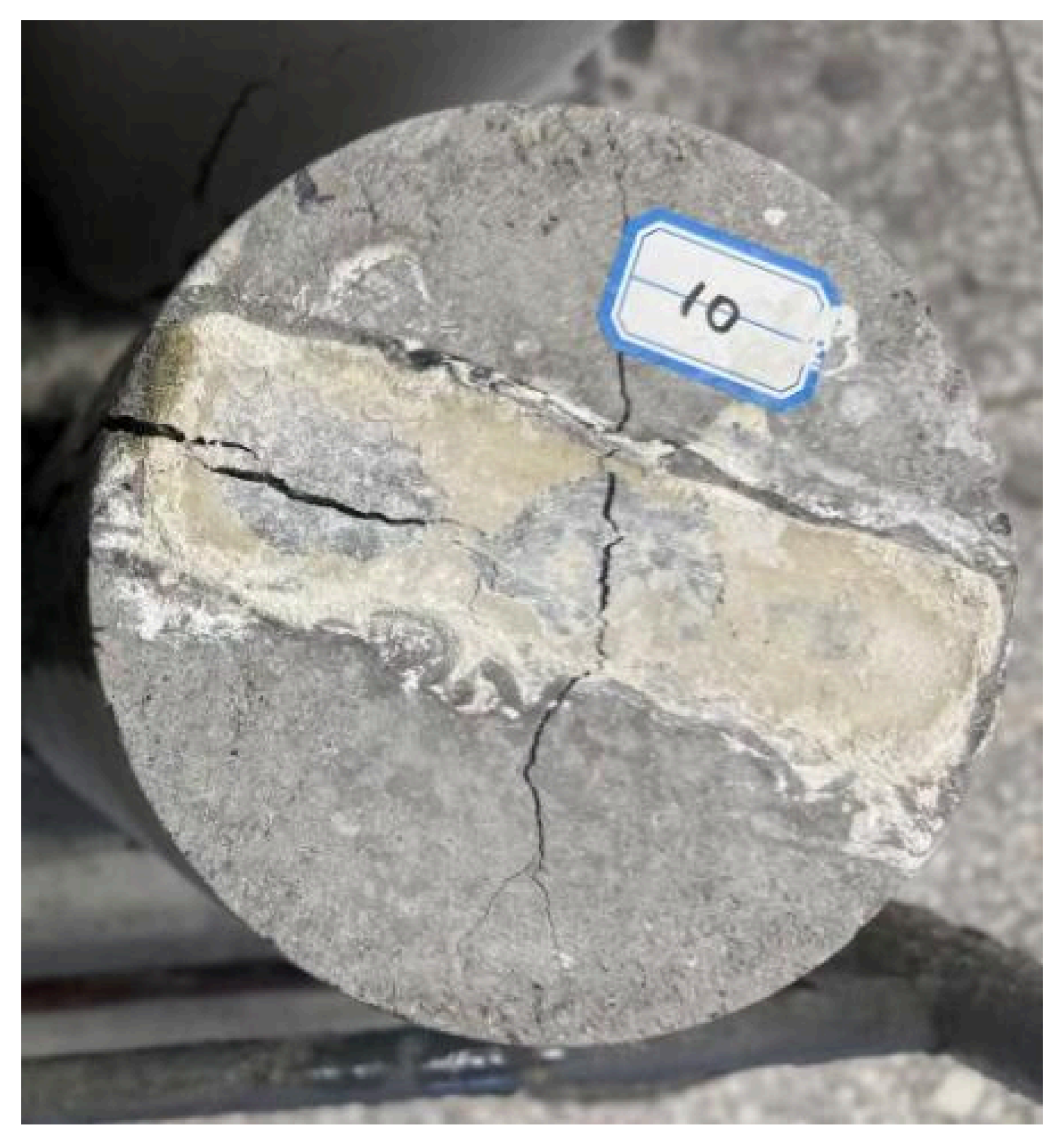
Failure mode of the concrete cylinder after testing.

Splitting tensile tests were performed in accordance with GB/T 50081-2019 [50] using a SYE-2000 testing machine. At the end of the 16-day MICP + WA treatment phase, specimens were placed horizontally and loaded diametrically, with the load applied parallel to the pre-existing crack plane (Figure 1b), perpendicular to the crack opening direction. A plywood bearing strip was placed along the full length of the cylinder to reduce stress concentration at the loading interface, as shown in Figure 5. Figure 6 shows the resulting failure mode. The splitting tensile strength $f_{\mathrm{ct}\;}$ was calculated by using Equation (1).

(1) $$f_{\mathrm{ct}\;}=\frac{2F}{\pi \;\times \;d\;\times \;L}$$

where $f_{\mathrm{ct}}$ is the split tensile strength (MPa); F is the maximum load at failure recorded (N)*;* d is the diameter of the specimen (mm); L is the length of the specimen (mm).

The tensile strength retention ratio (*R*_ct_) represents the repaired tensile strength relative to that of the intact specimen, highlighting the extent to which the original strength was retained after repair, which can be calculated using Equation (2).

(2) $$R_{\mathrm{ct}}=\frac{f_{\mathrm{ct},\;r\;}}{f_{\mathrm{ct},c}}\times 100\%$$

where $f_{\mathrm{ct},r}$ is the split tensile strength of repaired concrete specimens; $f_{\mathrm{ct},c}$ is that of the intact concrete specimens, all recorded in MPa, respectively.

#### 2.5.2. Capillary Water Absorption Test

**Figure 7:**
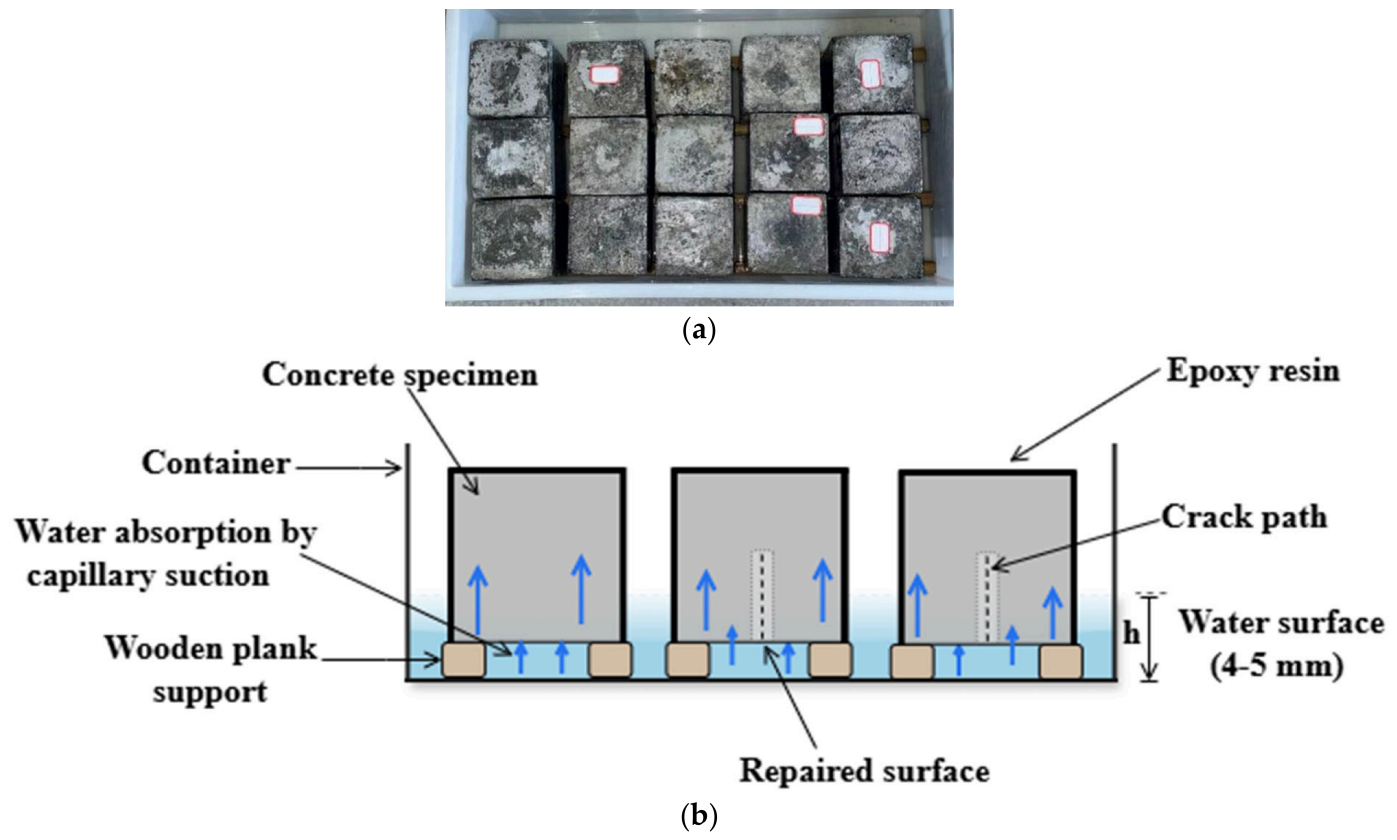
Water absorption test setup. (**a**) Concrete specimens submerged in water with wooden planks’ support. (**b**) Schematic diagram of the test setup with arrows indicating the capillary rise of water through the specimens. The mass was recorded at intervals of 1, 4, 6, 24, 48, and 76 h. Cumulative capillary water absorption was calculated in Equation (3):

With adjustments to specimen conditioning, capillary water absorption was measured in accordance with ASTM C1585 [51], where each specimen’s five faces were sealed with epoxy resin (Shanghai Duanjun Industrial Co., Ltd., Shanghai, China) (curing agent: resin ratio 1:4) and allowed to dry for 48 h. After that, specimens were oven-dried at 60 °C for at least three days until they reached a consistent mass. The exposed treated face was placed downward in contact with water at a depth of 4–5 mm, as shown in Figure 7. The schematic diagram in Figure 7b was drawn using draw.io (https://www.diagrams.net, accessed on 2 August 2026).

(3) $$i=\frac{m-m_{0}}{A\rho_{w}}=S\sqrt{t}+b$$

where i is the cumulative water absorption per unit area (mm); $m_{0}$ is the initial mass after drying (g); m is the mass after immersion at time t (g); A is the exposed surface area of the test face(mm^2^); $\rho_{w}$ is the density of water = 0.001 (g/mm^3^); S is the water absorption rate; $\sqrt{t}$ is the square root of time elapsed since the specimen first touched the water; b is the y-intercept.

At the largest crack width, the waterproof performance improvement coefficient ($\lambda_{w})$ was calculated to evaluate the repair effect of the concrete cubes in Equation (4).

(4) $$\lambda_{w}=\left|\frac{i_{r}-{\;i}_{u}}{i_{c}-{\;i}_{u}}\right|\times 100\%$$

where $i_{r}$ is the cumulative water absorption of the repaired specimens; $i_{u}$ is the cumulative water absorption of the cracked but unrepaired cracked specimen; $i_{c}$ is that of the intact specimen control, all in mm.

#### 2.5.3. Rapid Chloride Migration (RCM) Test

**Figure 8:**
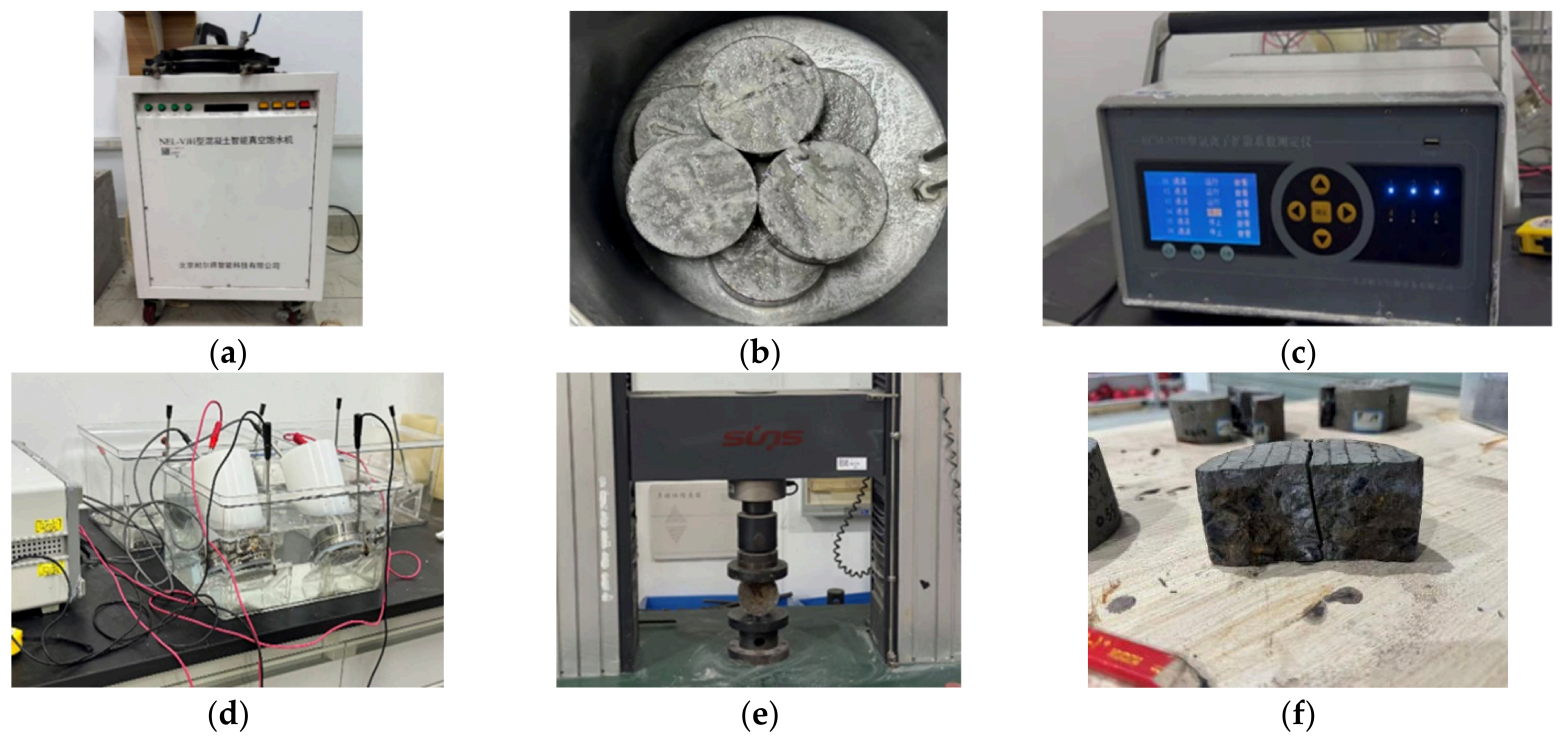
Rapid chloride migration test process. (**a**) Vacuum saturation machine. (**b**) Cylindrical specimens after the completion of saturation. (**c**) RCM test device. (**d**) Anode and cathode tank. (**e**) Specimen splitting into two. (**f**) Determination of penetration depth by colour change.

Before testing, specimens were vacuum-saturated (see Figure 8a) in a calcium hydroxide solution consisting of 20 g Calcium Oxide (CaO) in 15 L of water for approximately 48 h using a vacuum saturation machine (Model NEL-VJH, Beijing Nairede Intelligent Technology Co., Ltd., Beijing, China). Figure 8b shows specimens after the saturation was complete. Afterwards, each specimen was subjected to a voltage of 30 ± 0.2 V for 48 h using an RCM test device (Beijing Zhou’er Instrument Equipment Co., Ltd., Beijing, China) shown in Figure 8c, using 0.3 mol/L NaOH as the anolyte and 10% Sodium chloride (NaCl) as the catholyte (Figure 8d). Specimens were split perpendicular to the direction of the fracture after the test (see Figure 8e) using a UTM-305 universal testing machine (Shenzhen San-Si Zongheng Technology Co., Ltd., Shenzhen, China). A 0.1 mol/L AgNO_3_ solution was sprayed on them, and after 15 min, penetration depth was determined by the colour change, as shown in Figure 8f.

The split specimens’ chloride penetration depth (*X*_d_) was measured at seven points (*X*_1_–*X*_7_) spaced 10 mm apart. The chloride penetration depth ($X_{d}$) used in calculating the chloride migration coefficient *D*_RCM_ was the mean of measurements obtained from three specimens per group. The chloride migration coefficient was calculated by Equation (5) as follows:

(5) $$D_{RCM}=\;\frac{0.0239\left(273+T\right)L}{\left(U-2\right)t}\left(X_{d}-0.0238\sqrt{\frac{\left(273\;+\;T\right)LX_{d}}{U-2}}\;\;\right)$$

where $D_{\mathrm{RCM}}$ is the non-steady-state chloride migration coefficient (m^2^/s); T is the average (initial and final) anolyte solution temperature (°C); U is the applied voltage (V); t is the test duration (h); $X_{d}$ is the average value of the chloride penetration depth (mm) of the three specimens; L is the thickness of the specimen (50 mm).

The restoration efficiency of chloride resistance $\left(R_{\mathrm{Cl}}\right)$ was evaluated by comparing the intact samples (control) against the MICP-repaired specimens shown in Equation (6).

(6) $$\;R_{Cl}=\frac{D_{\mathrm{RCM},C}}{D_{\mathrm{RCM},\;R}}\times 100\%$$

where $D_{\mathrm{RCM},\;C}$ and $D_{\mathrm{RCM},\;R}$ represent the chloride migration in the intact concrete specimen and the chloride migration of the repaired specimen.

#### 2.5.4. Microstructural Analysis

Powder samples were collected from the fracture plane of split MICP + WA specimens after testing. X-ray diffraction analysis was used to determine mineral compositions of the tested samples and to identify crystalline phases present in the precipitate.

## 3. Results and Discussion

### 3.1. Crack Observations

**Figure 9:**
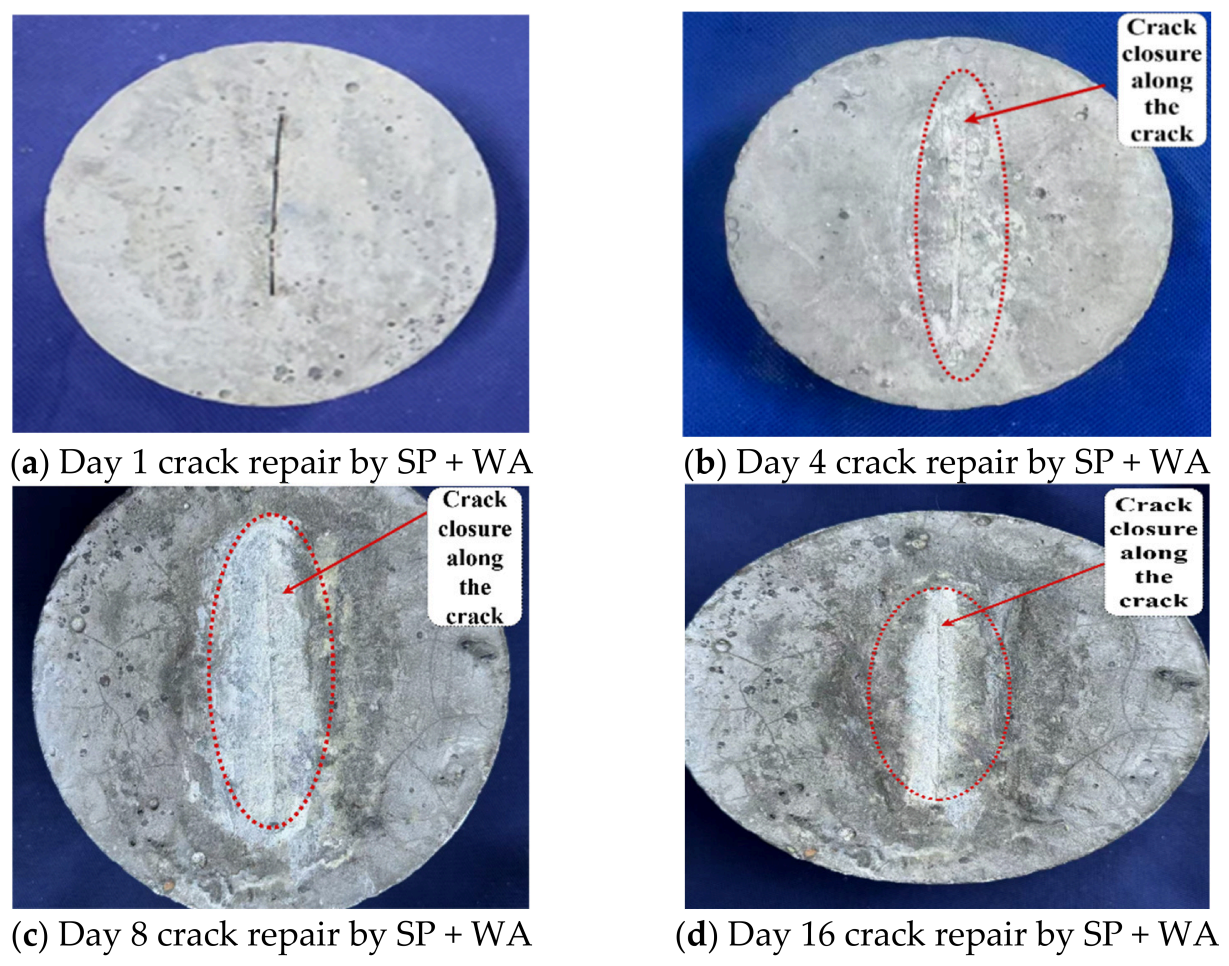
Crack sealing pattern over 16 days applying SP + WA. Note: Circled specimens indicated gradual crack closure pattern during the treatment period.

**Figure 10:**
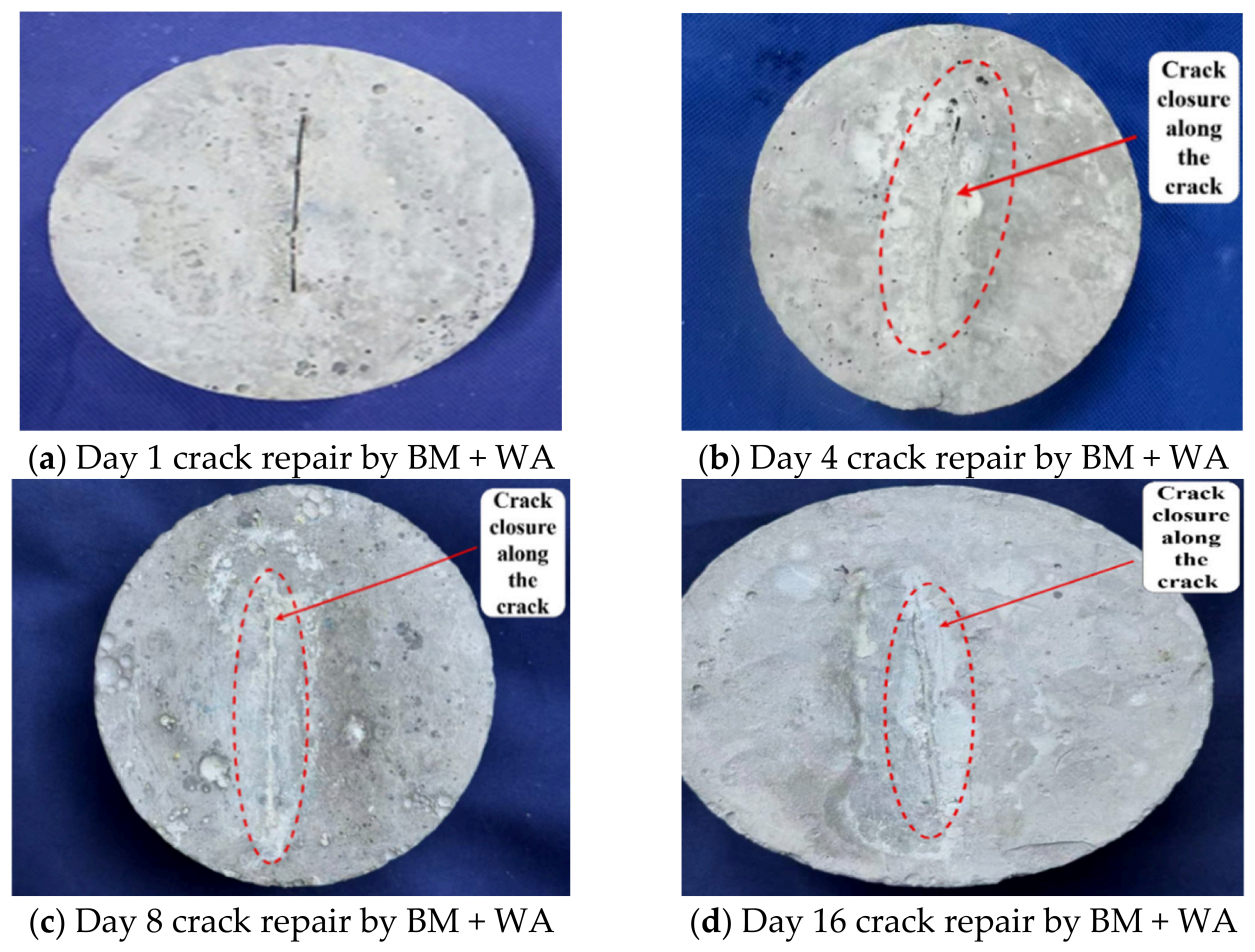
Crack sealing pattern over 16 days, applying BM + WA.

During the crack repair process, crack closure behaviour was monitored through periodic visual observation of the gradual formation of white precipitates around the repaired area. Figure 9 and Figure 10 show the gradual process of crack sealing during the 16-day repair period. Specimens of size 100 mm × 50 mm with a crack width of 0.4–0.5 mm were taken as an example to observe the gradual crack closure.

### 3.2. Split Tensile Strength Retention on Optimal Width and MICP Effectiveness

**Figure 11:**
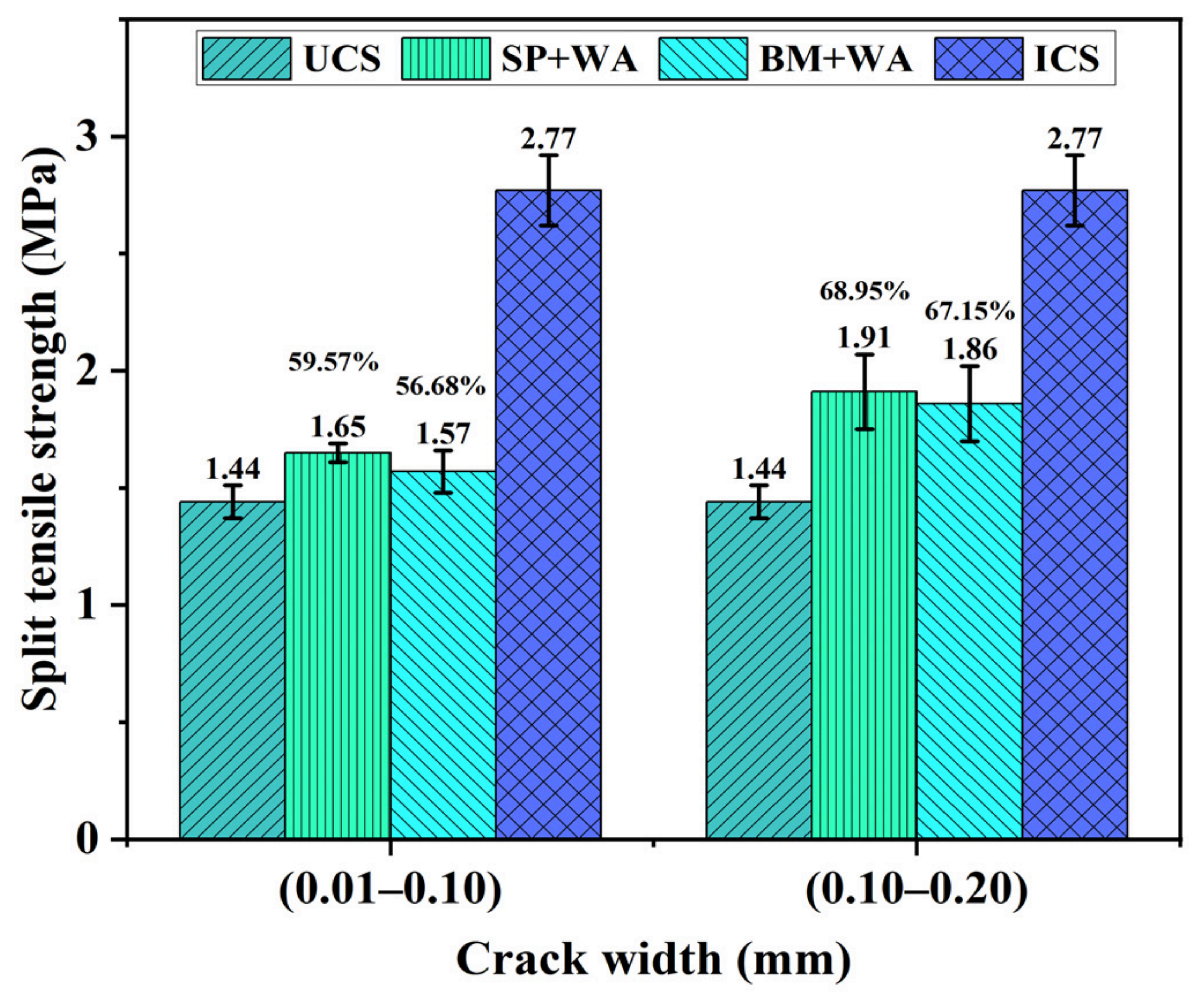
Comparison of split tensile strength across tested concrete cylinder specimens.

MICP + WA treatment improved the mechanical performance of cracked concrete relative to the unrepaired cracked specimens. The ICS attained a splitting tensile strength of 2.77 MPa as illustrated in Table 3 and Figure 11. The UCS averaged 1.44 MPa in tensile strength, showing a tensile strength loss of 48.01% of the original strength when compared to ICS, confirming that pre-existing cracks act as stress concentrators and significantly weaken load transfer across the crack plane.

For 0.05–0.10 mm, SP + WA and BM + WA achieved retention ratios of 59.57% and 56.68%, while for crack widths of 0.10–0.20 mm, 68.95 and 67.15% were achieved, respectively. Across both crack width ranges, the average tensile strength retention ratios relative to the ICS in SP + WA and BM + WA were 64.26% and 61.92%, respectively. The outcomes confirm that MICP + WA can partially restore tensile load transfer across crack interfaces. However, a tensile retention ratio of 68.95% in SP + WA compared to the intact specimen validates one of the limitations of the externally applied method, that the original cracked concrete cannot be reconstructed to its original form [52].

Most studies show an increase in tensile strength in narrower cracks, with recovery decreasing as the crack width increases. For example, past research by Lu et al. [38] reported recovery rates of 65.8% and 52.6% for crack widths of 0.05–0.10 mm and 0.10–0.30 mm. However, the present findings contradict previous findings, as narrower crack widths had lower recovery rates compared to wider crack widths. This may be because healing in cracks does not only rely on a large availability of calcites when additives are used. Considering dosages of colloidal thickeners based on the crack width range may help enhance penetration for calcium carbonates while avoiding blockage, thereby facilitating crack healing. The outcomes may also be attributed to the interaction between crack geometry and the MICP + WA system. Wider cracks provide more space for bacterial suspension retention and CaCO_3_ precipitation, which may facilitate calcite bridging across the crack plane, whereas narrower cracks may provide less space, limiting precipitation development within the crack. Also, variations in crack surface morphology and CaCO_3_ deposition may influence interface bonding and stress transfer efficiency after crack repair.

### 3.3. Capillary Water Absorption

**Figure 12:**
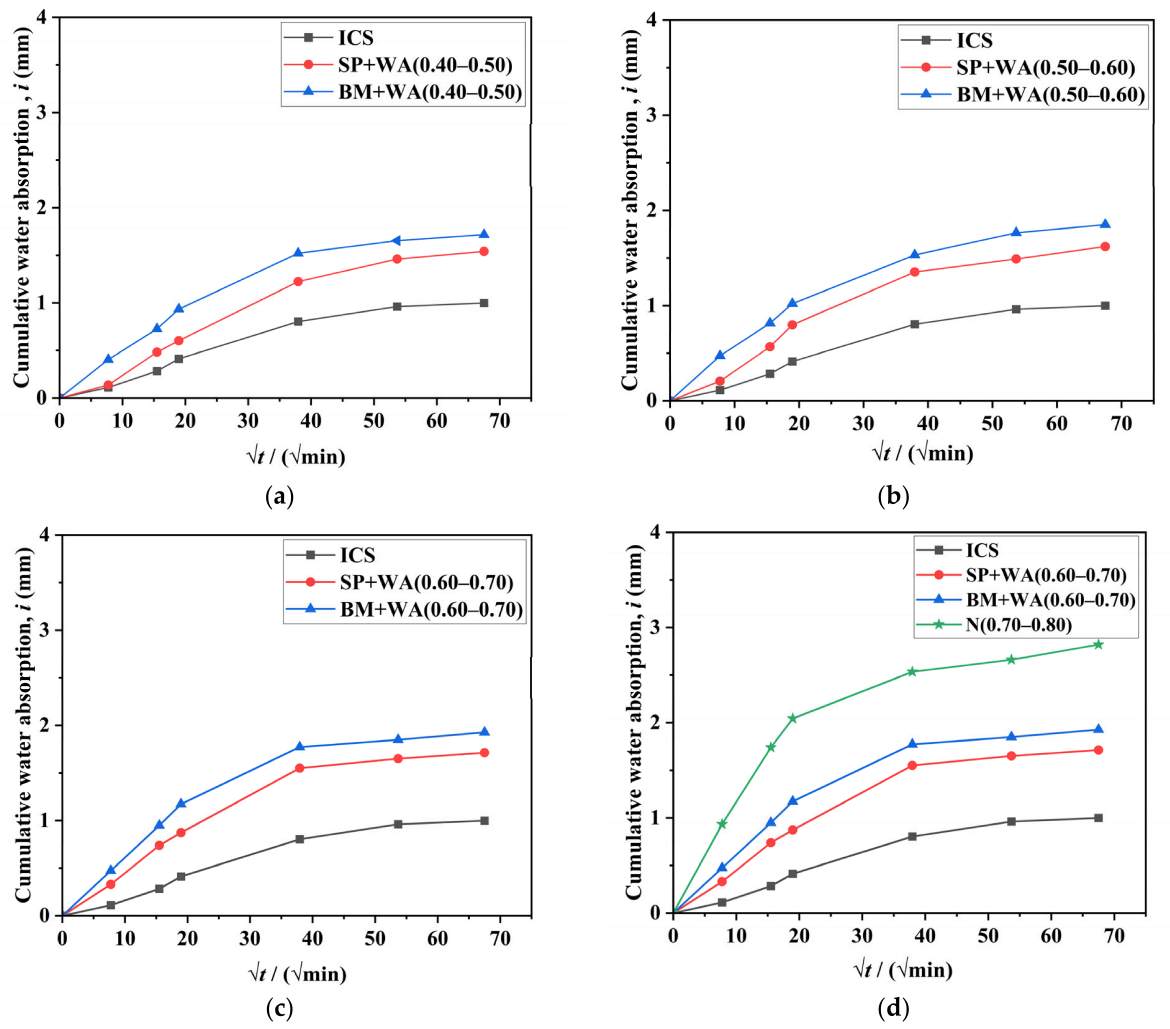
Cumulative water absorption height (*i*) with time. (**a**) Initial crack width (0.40–0.50 mm). (**b**) Initial crack width (0.50–0.60 mm). (**c**) Initial crack width (0.60–0.70 mm). (**d**) Initial crack width (0.60–0.70 mm) vs. UCS (0.70–0.80 mm).

Specimens exhibited a characteristic bilinear absorption pattern, consisting of a rapid initial water uptake stage (*t* < 6 h) followed by a slower secondary stage (*t* > 24 h). The initial absorption stage was controlled by capillary suction through interconnected pores and crack pathways, whereas the later stage reflected progressive saturation and a transition towards diffusion-dominated transport mechanisms [53,54]. As time increases, the water absorption rate decreases, following an inverse proportionality to $\sqrt{t}$, resulting in the linear relationship i vs. $\sqrt{t}$ (see Figure 12). This is consistent with the Lucas Washburn equation, which describes capillary-driven flow within the pore network [51,55]. Table 4 presents the mean values of the capillary water absorption height (mm) with respect to time measured at different intervals.

**Figure 13:**
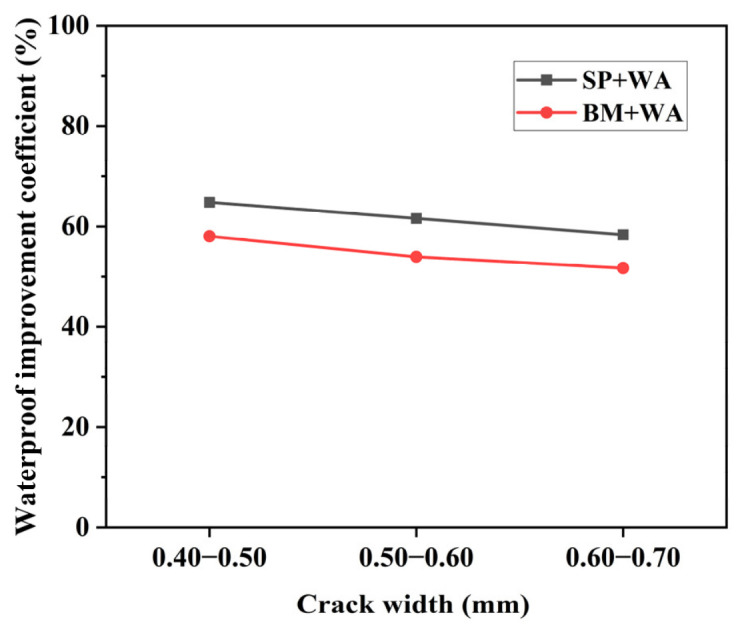
Waterproof performance improvement coefficient relative to ICS.

At $\sqrt{t}$ = 67.5 $\sqrt{\min\;}$, SP + WA achieved recovery efficiencies of 64.81%, 61.60%, and 58.36% for crack widths of 0.40–0.50 mm, 0.50–0.60 mm, and 0.60–0.70 mm, while BM + WA achieved recoveries of 58.02%, 53.95%, and 51.71%, respectively. A consistently higher recovery trend achieved by SP + WA suggests enhanced pore blocking and crack sealing compared to BM + WA, resulting in a greater reduction in moisture transport. It is observed that as crack width increases, recovery efficiency reduces, similar to previous studies [56,57,58]. This could be because, as cracks become wider, there is a gradual loss of capillary continuity across the crack location, since capillary water suction depends on an interconnected pore network for water ingress. Also, as the repair method transitions from injection to partial immersion at 0.60–0.70 mm, this trend may reflect the combined influence of crack width and application method. Figure 13 shows a decreasing trend in waterproof performance improvement as the crack width increases in repaired specimens.

At the widest crack width, SP + WA and BM + WA recorded mean absorption heights of 1.71 mm and 1.93 mm, representing reductions of 60.92% and 48.85%. These observations are consistent with prior research that found that MICP-based treatment can significantly reduce capillary water absorption across a wide range of crack widths, with notable improvements maintained even when cracks are not fully filled in a single repair cycle [59]. As established by the cubic law of fracture flow, the hydraulic conductance through a crack is proportional to the cube of its aperture [60], meaning even small increases in crack width lead to a disproportionate increase in water absorption.

### 3.4. Chloride Penetration Behaviour

**Figure 14:**
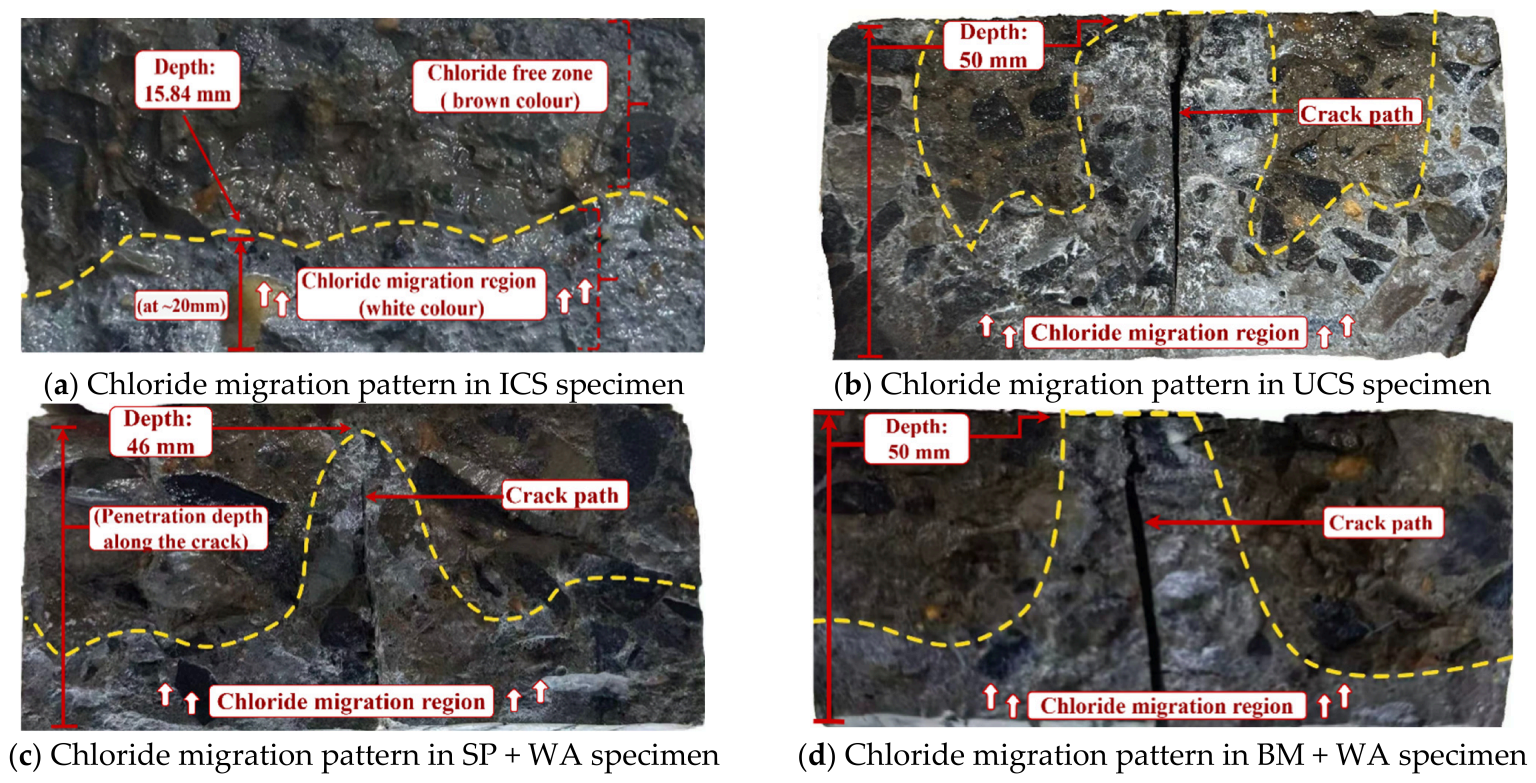
Qualitative assessment of spatial chloride migration patterns.

Figure 14 shows a colour change where the white colour indicates the presence of chloride ions, while the brown colour indicates a chloride-free region in the specimens. Table 5 presents the chloride penetration spatial depth outcomes and the means across all tested specimens.

**Figure 15:**
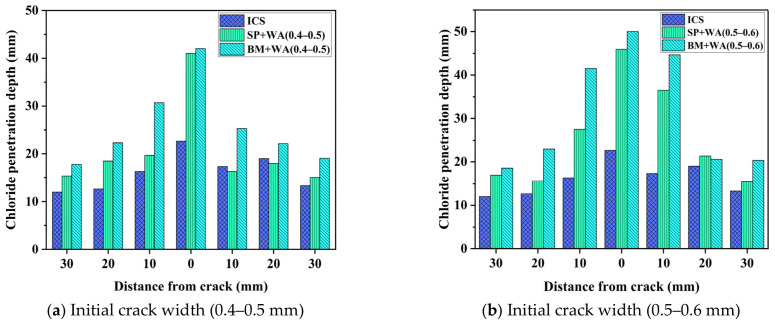

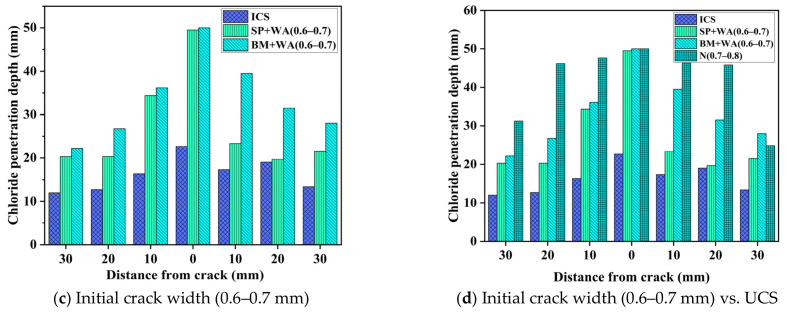
Spatial chloride penetration depth across all specimens.

Regarding the points away from the crack, the penetration depth was measured by means of three replicates. Evidently, the chloride penetration depth varied with crack width and distance from the crack location for both bacterial suspensions. As observed in Figure 15, the chloride penetration near the crack area is higher than in positions away from the crack. This may be because the crack plane acts as an ingress pathway for chloride ions along the crack area, regardless of the bacterial suspensions used. This is similar to a study by Yoon et al. [61], who identified that once a crack reaches a specific threshold, the crack site acts as a pathway for chloride ion transportation.

Comparing repaired specimens and the ICS, there is a gradual increase in penetration depth across all repaired specimens, highlighting the dominant influence of crack geometry on chloride transport. This is attributed to the volume of crack exceeding the amount of calcium carbonate formed during the 16-day repair cycle, leading to incomplete crack filling [62,63].

The chloride penetration behaviour pattern at different positions away from the crack location was analysed at different crack width ranges (0.4–0.5, 0.5–0.6, and 0.6–0.7 mm) between the two repaired groups, namely:

1. SP + WA-repaired specimens

Chloride penetration depths for SP + WA decreased progressively with distance from the crack: at ±10 mm, depths ranged from 18.51 to 32.02 mm, with the ICS at 16.83 mm. At ±20 mm, values narrowed to 18.25–20.00 mm, approaching the ICS value of 15.84 mm; and at ±30 mm, depths fell to 15.17–20.92 mm, with the ICS at 12.67 mm. SP + WA exhibited recovery rates of 82.1%, 69.3%, and 63.7%, respectively, with specimens at the narrowest crack width (0.4–0.5 mm) exhibiting chloride resistance approaching that of intact concrete.

2. BM + WA-repaired specimens

Higher penetration depths were recorded for BM + WA than for SP + WA at all measured distances: at ±10 mm, depths ranged from 27.97 to 43.09 mm; at ±20 mm, from 21.77 to 29.14 mm; and at ±30 mm, from 18.42 to 25.09 mm. BM + WA achieved chloride resistance recovery rates of 60.0%, 58.0%, and 50.5%, respectively, remaining consistently lower than SP + WA. For both bacterial systems, repair efficiency decreased with increasing crack width; at the narrowest crack width (0.4–0.5 mm), SP + WA exhibited better performance, with chloride penetration depths at ±30 mm approaching those of the ICS, suggesting enhanced chloride resistance away from the crack despite incomplete sealing at the crack vicinity.

Outcomes showed that chloride penetration is influenced by the closeness of the crack and the crack width. A high penetration depth is recorded at ±10 mm compared to the ICS, indicating an ingress pathway in the crack area for both bacterial suspensions. At ±20 mm and ±30 mm, penetration depths decreased with distance from the crack, indicating that the influence of cracking is localised. At ±30 mm, especially for SP + WA, the penetration depths relatively approach those of intact specimens, indicating that the concrete mass maintained its intrinsic resistance to chloride ingress, with deterioration localised near the crack location. Nevertheless, at the widest crack width tested, both repair systems outperformed the UCS, achieving chloride penetration reductions of 36.4% (SP + WA) and 20.8% (BM + WA), demonstrating retained resistance against chloride ingress.

### 3.5. Non-Steady-State Chloride Migration Coefficient (D_RCM_)

**Figure 16:**
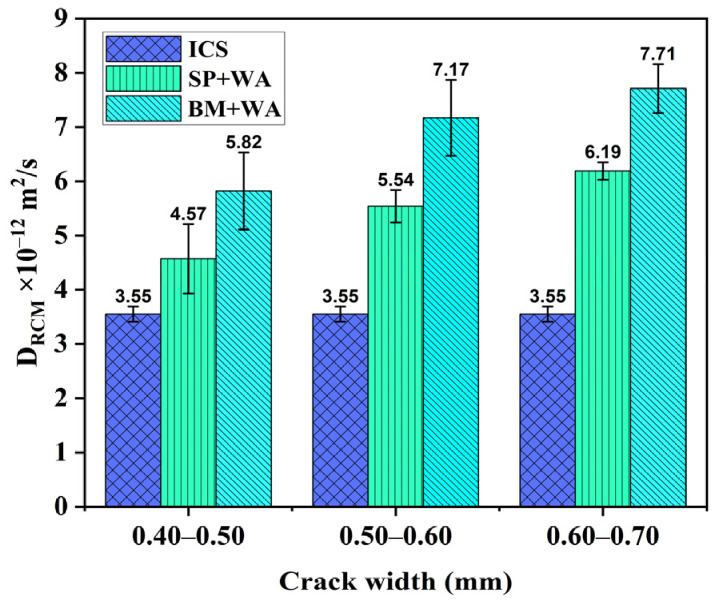
Non-steady-state chloride migration (*D*_RCM_) of each specimen group.

Figure 16 presents the *D*_RCM_ values across the repaired crack width groups. For instance, at a crack width range of 0.4–0.5 mm, SP + WA- and BM + WA-repaired specimens attained chloride migration coefficients (*D*_RCM_) of 4.57 and 5.82 × 10^−12^ m^2^/s, corresponding to chloride resistance recoveries of 77.68% and 61.00% relative to the ICS.

**Figure 17:**
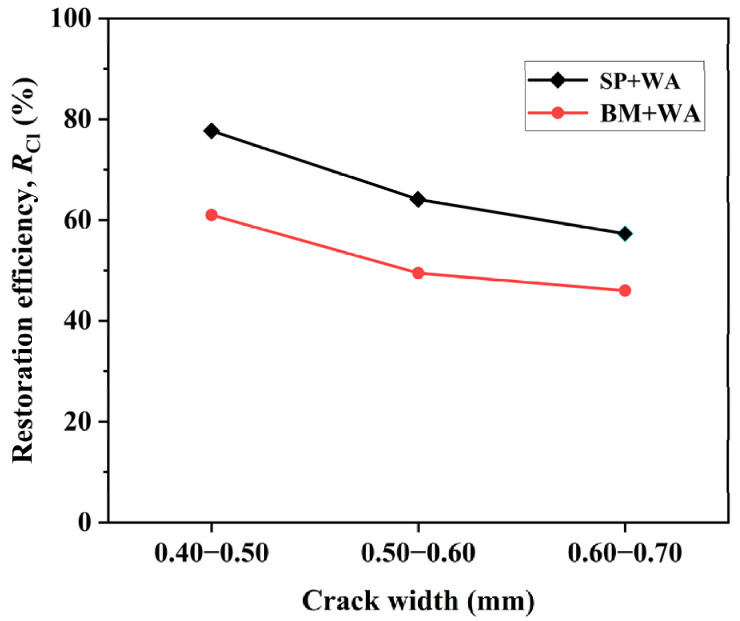
Restoration efficiency of chloride resistance (*R*_Cl_) relative to ICS.

As the crack width increased to 0.5–0.6 mm, the *D*_RCM_ values increased to 5.54 and 7.17 × 10^−12^ m^2^/s for SP + WA and BM + WA, respectively, resulting in reduced recovery rates of 64.08% and 49.51%. A similar trend was observed for crack widths of 0.6–0.7 mm, where SP + WA and BM + WA recorded *D*_RCM_ values of 6.19 and 7.71 × 10^−12^ m^2^/s, corresponding to recovery percentages of 57.35% and 46.04%, respectively, relative to the ICS. Figure 17 illustrates the corresponding restoration efficiency of chloride resistance percentages for all repaired specimens, showing a decrease in efficiency as the crack width increases.

A consistent pattern is observed that as crack width increases, $D_{\mathrm{RCM}}$ increases, indicating that there was partial healing of cracks in both treatment systems. As noted in Section 3.3, the transition from injection to partial immersion at 0.60 mm should be considered when interpreting this trend. Nevertheless, the continuous decline is consistent with the chloride penetration results in Figure 15. When partial sealing happens, this increases the chances of an effective chloride ion transportation, hence an increase in $D_{\mathrm{RCM}}$. The effectiveness of concrete repair by MICP + WA decreases when crack width increases, resulting in progressively less complete crack filling relative to the crack width within the repairing duration. This is similar to a study by Zou et al. (2024) [64], who presented a numerical model indicating that chloride resistance is directly proportional to the crack sealing by calcium carbonate infills. While both SP + WA and BM + WA repairs improve chloride resistance, particularly at narrower crack widths, crack width influences repair system effectiveness regardless of bacterial strain.

### 3.6. X-Ray Diffraction Analysis (XRD)

**Figure 18:**
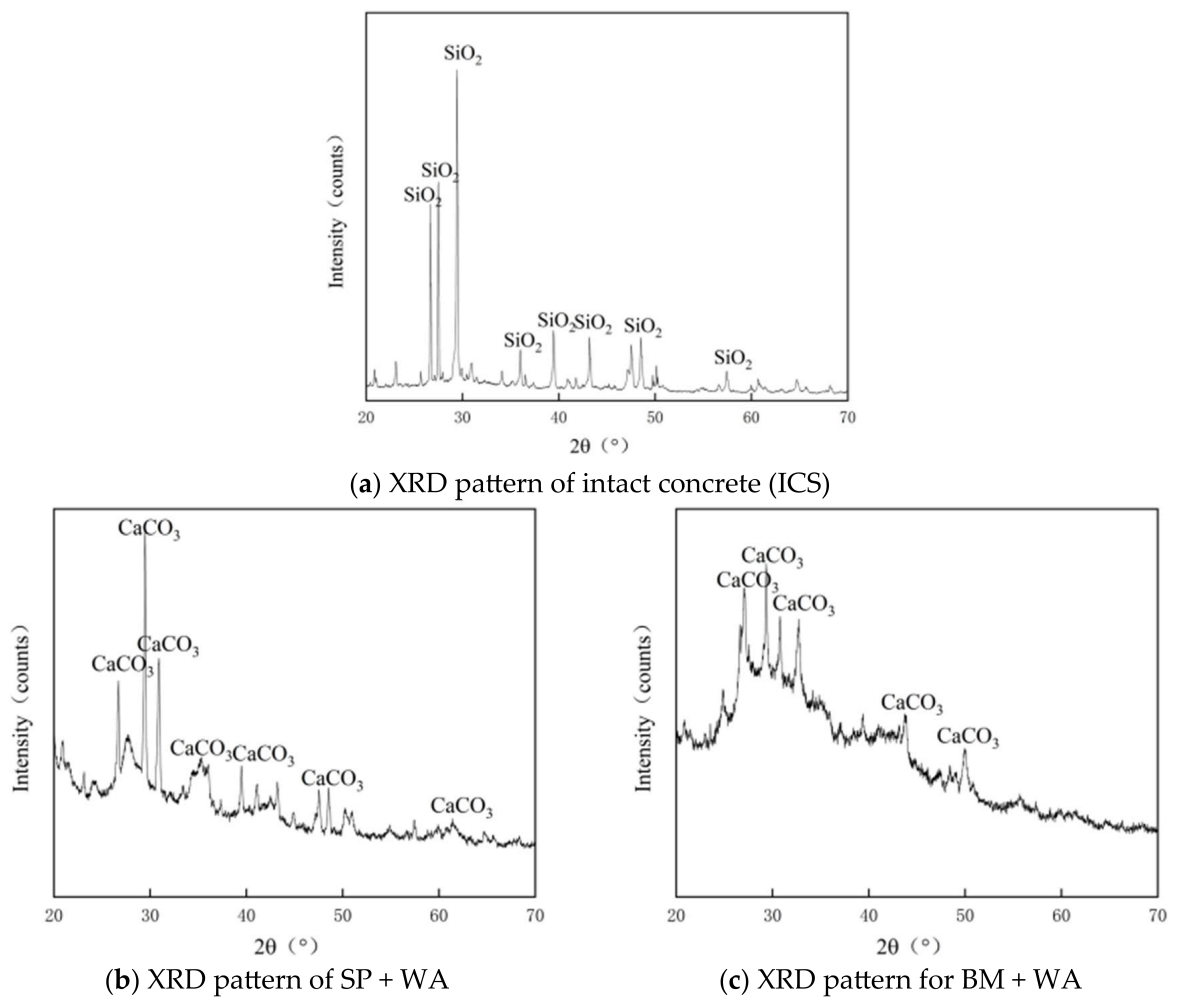
XRD patterns in identifying crystalline phases in ICS, SP + WA, and BM + WA.

XRD was performed on the precipitation deposits extracted from SP + WA and BM + WA to identify the crystalline phases in the ICS and MICP + WA-treated concrete. The resulting XRD patterns are illustrated in Figure 18, and the diffraction patterns were recorded over a 2θ (°) parameter.

The diffractive pattern for the ICS in Figure 18a exhibits only quartz (SiO_2_), validating that the concrete’s composition does not contain any detectable calcites, thereby establishing it as a baseline for comparison against MICP + WA repair groups. However, the patterns shown in Figure 18b,c confirmed that both SP + WA and BM + WA induced calcite precipitation (CaCO_3_), which was useful in the crack repairing employed in this study.

Nevertheless, the two bacterial suspensions show variations in peak intensity and sharpness, suggesting a substantial difference in calcite characteristics of the formed CaCO_3_. The XRD pattern of SP + WA exhibited steeper and sharper peaks, whereas BM + WA showed wider and fewer peaks, suggesting a scattered nanocrystalline CaCO_3_ structure. These observations align with previous studies, which have shown that ureolytic activity is associated with high peaks in the polymorph, while carbonic anhydrase pathways tend to produce more scattered precipitates [65,66]. However, because the two systems employed different cementation chemistries, the observed crystallinity differences in the polymorph may reflect the combined influence of metabolic pathway and cementation environment rather than bacterial metabolism alone.

The absence of vaterite on the polymorph, commonly detected in MICP processes using calcium acetate as the calcium source, may be due to the stabilising influence of Welan gum and Attagel 50. The use of both additives increases suspension and viscosity, hence stabilising the suspensions and promoting calcite formation while suppressing metastable polymorphs.

## 4. Conclusions

This study incorporated Welan gum and Attagel 50 as viscosity-modifying agents (VMAs) within microbially induced calcium carbonate precipitation (MICP) technology and compared the performance of ureolytic *Sporosarcina pasteurii* (SP + WA) and non-ureolytic *Bacillus mucilaginosus* (BM + WA) in repairing cracked concrete. Strength, durability, and microstructural tests were used to assess crack healing performance, permeability reduction, and precipitation characteristics. The main conclusions are as follows:

1. Crack Repair and Mechanical Performance: Pre-induced cracks showed partial tensile strength recovery following MICP + WA repair. At crack widths of 0.05–0.10 mm, SP + WA and BM + WA achieved tensile strength retention ratios of 59.57% and 56.68%, increasing to 68.95% and 67.15% at 0.10–0.20 mm. Overall, average retention ratios of 64.26% and 61.92% confirmed partial restoration of mechanical integrity. The higher recovery at wider crack widths may be attributed to increased void space for bacterial suspension retention and CaCO_3_ precipitation, whereas narrower crack widths may restrict precipitation development and interface bonding, resulting in reduced repair efficiency.
2. Permeability Behaviour: Capillary water absorption exhibited an initial rapid suction phase followed by a gradual reduction over time, with recovery efficiency trends of 64.81%, 61.60%, and 58.36% for SP + WA; 58.02%, 53.95%, and 51.71% for BM + WA relative to the ICS at crack widths of 0.4–0.5, 0.5–0.6, and 0.6–0.7 mm, respectively, with performance decreasing as crack width increased. At the widest crack width tested, waterproof improvement coefficients of 60.92% and 48.85% for SP + WA and BM + WA were achieved, confirming reduced water absorption.
3. Chloride Migration Behaviour: Although all repaired specimens showed higher chloride penetration near the crack location than the ICS, penetration depth decreased with distance from the crack and approached that of intact concrete at approximately ±30 mm. Chloride resistance recoveries reached 82.1%, 69.3%, and 63.7% for SP + WA, while BM + WA achieved 60.0%, 58.0% and 50.5%. For *D*_RCM_, recoveries of 77.68%, 64.08%, and 57.35% were achieved for SP + WA, whereas BM + WA achieved 61.00%, 49.51%, and 46.04%, respectively. At the widest crack width, both bacterial suspensions outperformed the unrepaired specimen, reducing chloride penetration by 36.4% and 20.8%.
4. Microstructural Evidence and Comparative Performance: X-ray diffraction confirmed calcite as the sole detectable CaCO_3_ polymorph in both SP + WA- and BM + WA-treated specimens. SP + WA exhibited sharper, high-intensity peaks indicating differences in calcite crystal characteristics, whereas BM + WA showed broader peaks. The absence of vaterite may be attributed to the stabilising effect of Welan gum and Attagel 50, which promoted preferential calcite formation while suppressing metastable polymorphs.

In conclusion, the SP + WA and BM + WA repairs were effective in improving mechanical performance and short-term durability, as reflected by transport resistance under the controlled laboratory conditions adopted in this study, with SP + WA exhibiting better performance trends than BM + WA across all measured parameters. The outcomes also indicate that, within each induction method, crack width is a factor in repair system efficiency, as increasing crack width reduces crack sealing effectiveness and consequent performance recovery.

While the present findings demonstrate the potential of MICP + VMA for crack repair and durability enhancement, several limitations should be noted. The study examined only the combined MICP + WA at a single VMA dosage and lacked MICP-only, WA-only, or cementation solution-only controls, precluding quantitative differentiation between the contributions of MICP and the VMA carrier system’s physical and rheological effects. Raw replicate values for the capillary water absorption dataset were not retained, preventing the calculation of dispersion; the SP + WA and BM + WA comparisons were interpreted as a descriptive trend. Long-term durability under freeze–thaw, wetting–drying, carbonation, and acidic exposure was not assessed; therefore, the durability findings reported in this study are limited to controlled laboratory conditions. Moreover, the 16-day treatment period may also limit field applicability due to the challenges associated with extended treatment implementation. Future work should compare VMA and non-VMA systems for quantification, refine suspension dosage considering crack width to improve penetration and crack healing efficiency, assess suitable treatment duration, and evaluate long-term performance under realistic cracking and exposure conditions for field applications.

## Figures and Tables

**Table 1:** Concrete mix ratio.

w/c Ratio	Cement (kg/m^3^)	Water (kg/m^3^)	Sand (kg/m^3^)	Gravel (kg/m^3^)
0.49	449	220	615	1116

**Table 2:** Summary of concrete specimens.

Specimen Shape	Specimen Size (mm)	Method of Crack Induction	Crack Width (mm)	Number of Specimen Groups	Repair Method	Use
Cylinder	150 × 300	N/A Mechanical loading	N/A 0.05–0.10 0.10–0.20 0.10–0.20	1 2 2 1	N/A Partial immersion Partial immersion N/A	Split tensile testing
Cube	100 × 100 × 100	N/A Steel Plate	N/A 0.40–0.50 0.50–0.60 0.60–0.70 0.70–0.80	1 2 2 2 1	N/A Injection Injection Partial immersion N/A	Water absorption test
Cylinder	100 × 50	N/A Steel Plate	N/A 0.40–0.50 0.50–0.60 0.60–0.70 0.70–0.80	1 2 2 2 1	N/A Injection Injection Partial immersion N/A	Rapid chloride migration test

Note: Each specimen group consists of three specimens (*n* = 3).

**Table 3:** Tensile strength test results.

Group	Crack Width Value (mm)	Crack Width Range (mm)	Tensile Strength (MPa)			Mean Tensile Strength (MPa)	SD (MPa)	CoV
			1st	2nd	3rd			
ICS	N/A	N/A	2.63	2.94	2.73	2.77	0.15	0.05
UCS	0.147; 0.14; 0.152	0.10–0.20	1.42	1.51	1.39	1.44	0.07	0.05
SP + WA	0.196; 0.105; 0.161	0.10–0.20	1.95	1.73	2.04	1.91	0.16	0.08
BM + WA	0.196; 0.105; 0.112	0.10–0.20	2.01	1.70	1.87	1.86	0.16	0.08
SP + WA	0.084; 0.098; 0.077	0.05–0.10	1.61	1.67	1.67	1.65	0.04	0.02
BM + WA	0.084; 0.091; 0.063	0.05–0.10	1.49	1.55	1.67	1.57	0.09	0.06

**Table 4:** Water absorption height with respect to time.

Specimen Group (Crack Width, mm)	Capillary Water Absorption Height, i (mm) at $\sqrt{t}\;(\sqrt{\min})$					
	7.75	15.49	18.97	37.95	53.67	67.53
ICS	0.111	0.283	0.411	0.918	0.951	0.998
SP + WA (0.4–0.5)	0.137	0.482	0.602	1.223	1.483	1.535
BM + WA (0.4–0.5)	0.404	0.727	0.933	1.521	1.653	1.717
SP + WA (0.5–0.6)	0.206	0.567	0.797	1.353	1.489	1.620
BM + WA (0.5–0.6)	0.472	0.815	1.021	1.531	1.765	1.850
SP + WA (0.6–0.7)	0.329	0.739	0.873	1.551	1.650	1.712
BM + WA (0.6–0.7)	0.473	0.949	1.173	1.773	1.848	1.927
N (0.7–0.8)	0.933	1.740	2.043	2.534	2.661	2.819

Note: All cumulative capillary water absorption height values (mm) are presented as mean values (*n* = 3). Estimates of dispersion (S.D and CoV) could not be calculated owing to the unavailability of raw replicate data. The absence of variability metrics limits statistical comparisons; therefore, the SP + WA and BM + WA comparison was interpreted as a descriptive trend rather than a statistically confirmed difference.

**Table 5:** Chloride penetration spatial depth across all specimens.

Specimen Group and Crack Width Range (mm)	Chloride Penetration Depth $X_{d}\;$(mm)			Mean $X_{d}$ (mm)	SD ($X_{d}$) (mm)	CoV
	**1st**	**2nd**	**3rd**			
ICS SP + WA (0.4–0.5) BM + WA (0.4–0.5)	16.86 17.43 28.57	16.00 22.07 22.83	15.71 22.14 25.43	16.19 20.55 25.61	0.60 2.70 2.88	0.04 0.13 0.11
SP + WA (0.5–0.6) BM + WA (0.5–0.6) SP + WA (0.6–0.7) BM + WA (0.6–0.7) UCS (0.7–0.8)	26.00 28.43 26.43 32.00 40.07	26.43 30.83 27.71 32.57 44.41	24.47 34.43 26.86 35.74 39.96	25.63 31.23 27.00 33.43 41.48	1.03 3.02 0.65 2.02 2.54	0.04 0.10 0.02 0.06 0.06

## Data Availability

The original contributions presented in this study are included in the article. Further inquiries can be directed to the corresponding author.
